# Distinct Osteogenic Potentials of BMP-2 and FGF-2 in Extramedullary and Medullary Microenvironments

**DOI:** 10.3390/ijms21217967

**Published:** 2020-10-27

**Authors:** Shuji Nosho, Ikue Tosa, Mitsuaki Ono, Emilio Satoshi Hara, Kei Ishibashi, Akihiro Mikai, Yukie Tanaka, Aya Kimura-Ono, Taishi Komori, Kenji Maekawa, Takuo Kuboki, Toshitaka Oohashi

**Affiliations:** 1 Department of Molecular Biology and Biochemistry, Okayama University Graduate School of Medicine, Dentistry and Pharmaceutical Sciences, Okayama 700-8558, Japan; de422038@s.okayama-u.ac.jp (S.N.); p9po6y8u@s.okayama-u.ac.jp (K.I.); a.mikai@s.okayama-u.ac.jp (A.M.); pblq4l6l@s.okayama-u.ac.jp (Y.T.); oohashi@cc.okayama-u.ac.jp (T.O.); 2 Department of Oral Rehabilitation and Regenerative Medicine, Okayama University Graduate School of Medicine, Dentistry and Pharmaceutical Sciences, Okayama 700-8558, Japan; de421035@s.okayama-u.ac.jp (I.T.); a-kimura@md.okayama-u.ac.jp (A.K.-O.); de19016@s.okayama-u.ac.jp (T.K.); maekawa@md.okayama-u.ac.jp (K.M.); kuboki@md.okayama-u.ac.jp (T.K.); 3 Department of Biomaterials, Okayama University Graduate School of Medicine, Dentistry and Pharmaceutical Sciences, Okayama 700-8558, Japan; gmd421209@s.okayama-u.ac.jp; 4 Center for Innovative Clinical Medicine, Okayama University Hospital, Okayama 700-8558, Japan

**Keywords:** BMP-2, FGF-2, bone formation, bone marrow

## Abstract

Bone morphogenetic protein-2 (BMP-2) and fibroblast growth factor-2 (FGF-2) have been regarded as the major cytokines promoting bone formation, however, several studies have reported unexpected results with failure of bone formation or bone resorption of these growth factors. In this study, BMP-2 and FGF-2 adsorbed into atellocollagen sponges were transplanted into bone defects in the bone marrow-scarce calvaria (extramedullary environment) and bone marrow-abundant femur (medullary environment) for analysis of their in vivo effects not only on osteoblasts, osteoclasts but also on bone marrow cells. The results showed that BMP-2 induced high bone formation in the bone marrow-scarce calvaria, but induced bone resorption in the bone marrow-abundant femurs. On the other hand, FGF-2 showed opposite effects compared to those of BMP-2. Analysis of cellular dynamics revealed numerous osteoblasts and osteoclasts present in the newly-formed bone induced by BMP-2 in calvaria, but none were seen in either control or FGF-2-transplanted groups. On the other hand, in the femur, numerous osteoclasts were observed in the vicinity of the BMP-2 pellet, while a great number of osteoblasts were seen near the FGF-2 pellets or in the control group. Of note, FCM analysis showed that both BMP-2 and FGF-2 administrated in the femur did not significantly affect the hematopoietic cell population, indicating a relatively safe application of the two growth factors. Together, these results indicate that BMP-2 could be suitable for application in extramedullary bone regeneration, whereas FGF-2 could be suitable for application in medullary bone regeneration.

## 1. Introduction

Large bone defects caused, for instance, by tumor, trauma, or infection, induce substantial functional impairment and consequent esthetic and psychological distress for the patients. Nevertheless, reconstruction and regeneration of such large bone defects are yet a significant challenge in orthopedic and oral and maxillofacial fields.

Bone grafting is a surgical procedure that promotes the reconstruction of large bone defects with materials, among which autogenous bone grafts have long been considered as the gold standard and the most effective material [1,2]. However, autogenous bone grafts present significant drawbacks, such as the risk of infection at the donor site and the restricted amount of harvestable bone [3,4]. Thus, a number of bone substitute materials have been developed to overcome the problems associated with autogenous bone graft using cells (e.g., bone marrow-derived stem cells or adipose-derived stem cells) or materials [e.g., collagen, *β*-tricalcium phosphate (*β*-TCP), hydroxyapatite (HA)] with/without a combination of cytokines [e.g., fibroblast growth factors (FGFs), bone morphogenetic proteins (BMPs) and platelet-derived growth factor (PDGF)] [5,6,7,8]. Among several cytokines, FGF-2 and BMP-2 are of particular interest because they are currently widely utilized in periodontal surgery and spine fusion or oral-maxillofacial surgery, respectively [9,10,11,12].

The FGF family is a group of multifunctional cytokines that regulate a number of complex biological processes related to tissue development and homeostasis [13,14]. In bone, FGF signaling has key roles in regulating osteogenesis and mineral homeostasis [14,15]. Among FGFs, FGF-2 is one of the most effective cytokines that promote angiogenesis and osteogenesis and recruitment of mesenchymal stem cells and osteoblasts [16].

BMPs are growth factors that belong to the transforming growth factor (TGF)-*β* superfamily and have various functions in embryonic development and patterning, tissue homeostasis, and organ regeneration [17,18,19]. Many of the BMPs are characterized by the ability to induce ectopic and orthotopic bone formation [20,21,22], particularly, BMP-2 is well known to be a strong inducer of bone formation and to play important roles in the development and regeneration of bone and cartilage [17,18,23]. In 2003, the US Food and Drug Administration (FDA) approved the application of recombinant human BMP-2 (rhBMP-2) for the anterior lumbar interbody fusion, and currently, rhBMP-2 is widely used in spine and oral-maxillofacial surgeries [9,10,12].

Together, rhFGF-2 and rhBMP-2 could be the major cytokines for application in regenerative therapies of large bone defects [9,10,11,12]. However, an increasing number of studies have reported complications, such as failure of bone regeneration and bone resorption, associated with the clinical use of the two growth factors [24].

Recently, our research group also reported that rhBMP-2 transplanted in the bone marrow-abundant environment induced bone resorption in a canine dental implant model [25]. On the other hand, Nagayasu et al. showed that bone formation was accelerated by the administration of FGF-2 in the bone marrow environment [26]. From these reports, we hypothesized that the bone-forming capabilities of FGF-2 and BMP-2 largely depend on the presence or absence of a bone marrow environment at the recipient site, and that elucidation of the mechanisms of FGF-2 and BMP-2 functions at these different sites would be the key for not only the successful clinical treatments using rhFGF-2 and rhBMP-2, but also the development of novel composite biomaterials that can optimize the function of the two growth factors.

Therefore, the aim of this study was to evaluate the effect of FGF-2 and BMP-2 on bone regeneration in both bone marrow scarce or abundant environments at the tissue level, as well as at the cellular level, by investigating their effects on various cells present in the bone marrow, such as osteoblasts, osteoclasts, mesenchymal cells, hematopoietic cells and endothelial cells. The results demonstrated that BMP-2 promoted higher bone formation than FGF-2 in bone marrow-scarce calvaria, but the opposite effect was observed in marrow-abundant femurs.

## 2. Results

### 2.1. BMP-2, but Not FGF-2, Promotes the Repair of Mouse Calvarial Defect

First, in order to investigate the ability of FGF-2 and BMP-2 to induce bone regeneration in the bone marrow-scarce calvaria, freeze-dry atellocollagen pellets containing 1 or 10 μg of FGF-2 or 10 μg of BMP-2 were transplanted into mouse calvarial bone defects. Because Charoenlarp et al. have reviewed that a microgram dose of FGF-2 could induce bone formation in rodents [16], concentrations of 1 and 10 μg of FGF-2 were used in this study. On the other hand, since our previous analysis showed that BMP-2 (1, 10, and 100 μg) dose-dependently enhanced bone formation in mouse calvarial and inhibited bone formation in mouse femoral bone defects [25], a single dose of BMP-2 (10 μg) was used in this study.

**Figure 1:**
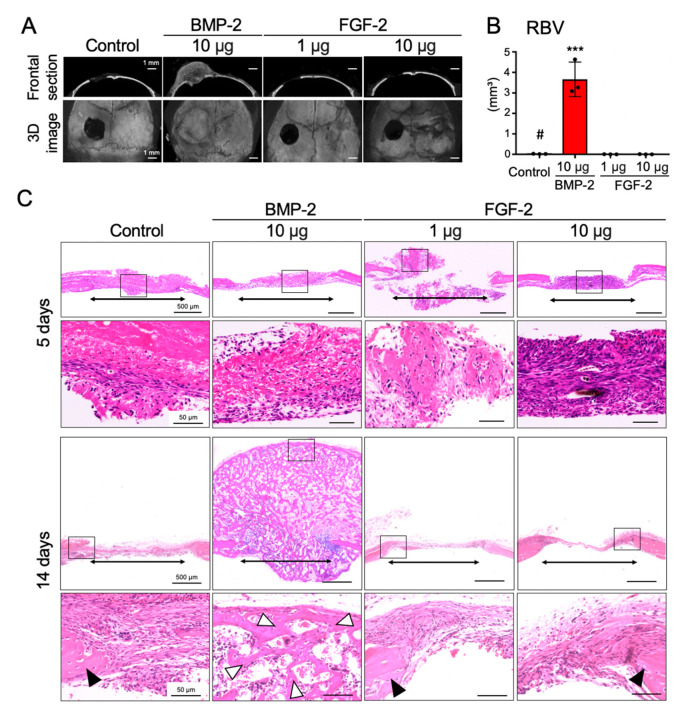
BMP-2, but not FGF-2, promotes repair of mouse calvarial defect. Collagen pellets containing BMP-2 (10 µg) or FGF-2 (1 µg, 10 µg) or distilled water (DW, control) were transplanted into mouse calvarial defects. (**A**) Frontal section (upper panel) and 3D (lower panel) images of micro-CT, 14 days after transplantation. (**B**) Graph shows the quantitative analysis of the regenerated bone volume (RBV) (*n* = 3, *** *p* < 0.001 versus control pellet (#). One-way ANOVA/Tukey). (**C**) HE-staining of frontal sections at the defect area at 5 days and 14 days after surgery. The results are representative of at least three independent experiments. Lower panels are high magnification images of the squares in the upper images. Two-way arrows, white arrowheads and black arrowheads indicate the bone defects, newly-formed bone and calvarial bone, respectively.

Quantitative analysis of the regenerated bone volume (RBV) using micro-CT showed that 10 μg of BMP-2 significantly induced bone formation compared with the control group, 14 days after transplantation. However, bone formation was not observed in the groups transplanted with 1 μg and 10 μg of FGF-2 (Figure 1A,B). In accordance with the results of the micro-CT analysis, histological investigations revealed the detailed aspect of the newly regenerated bone formed only in the BMP-2-transplanted group at 14 days post-transplantation, but bone formation was not observed in any group at 5 days post-transplantation (Figure 1C).

### 2.2. FGF-2, but Not BMP-2, Promotes the Repair of Mouse Femoral Defect

**Figure 2:**
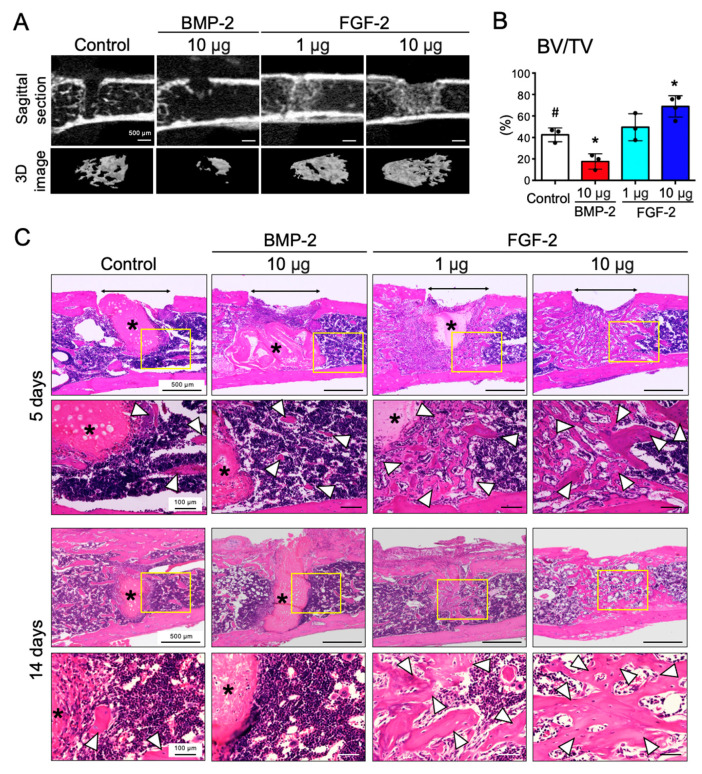
FGF-2, but not BMP-2, promotes repair of mouse femoral defect. Collagen pellets containing BMP-2 (10 µg) or FGF-2 (1 µg, 10 µg) or DW (control) were transplanted into mouse femur defects. (**A**) Micro-CT sagittal images of the femur (upper panel) and 3D reconstructed images of the trabecular bone (lower panel) of an area ranging from 1 mm above and below the defect. (**B**) Graph shows the quantitative analysis of BV/TV of an area within 1 mm above and below the defect in the femur. (*n* = 3–4, * *p* < 0.05 versus control pellet (#). One-way ANOVA/Tukey). (**C**) HE-staining of frontal sections at the femur defect area at 5 days and 14 days post-surgery. The results are representative of at least three independent experiments. Lower panels are high magnification images of the squares in the upper images (remained collagen pellet *). Two-way arrows and white arrowheads indicate the bone defects and newly-formed bone, respectively.

Next, to examine the effects of FGF-2 and BMP-2 in the bone marrow-abundant environment, pellets containing 1 or 10 μg of FGF-2 or 10 μg of BMP-2 were transplanted into mouse femoral defects. Micro-CT analysis showed that the trabecular bone in the marrow was resorbed by BMP-2 administration, as reported previously [25]. Interestingly, however, FGF-2 significantly promoted bone formation in the marrow in a dose-dependent manner (Figure 2A). The results of the quantitative analysis confirmed the opposing effects of BMP-2 and FGF-2 in inducing bone formation in the marrow environment (Figure 2B).

Figure 2C shows the images of HE stained femurs after 5 and 14 days of transplantation. Although bony areas were observed around the transplanted pellets in the control and BMP-2 groups at 5 days post-transplantation, no mature bone was observed around the BMP-2 pellets after 14 days of transplantation. In the groups transplanted with FGF-2, bone formation was observed at the implanted site both at 5 days and 14 days post-transplantation.

### 2.3. Depletion of Bone Marrow Cells Inhibits FGF-2-Induced Bone Formation in the Marrow Cavity

**Figure 3:**
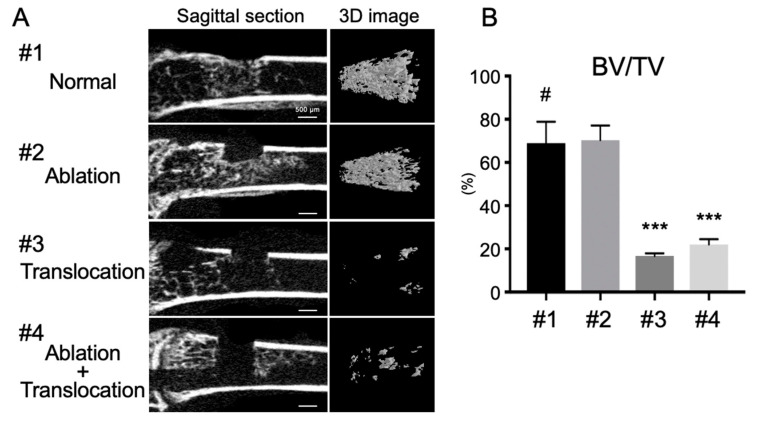
Depletion of bone marrow cells inhibits FGF-2-induced bone formation in the marrow cavity. FGF-2-adsorbed collagen pellets were implanted in mouse femur defects in the following conditions: (#1) Intact marrow (normal, control); (#2) Ablated marrow (ablation); (#3) Femur translocation; (#4) Marrow ablation and femur translocation. (**A**) Sagittal section images of femur defect areas (left panel) and 3D images of the trabecular bone (right panel). (**B**) Quantitative evaluation of BV/TV of trabecular bone in femur defect area. (*n* = 4, *** *p* < 0.001 versus control pellet (#). One-way ANOVA/Tukey).

Previously, our group has reported that the number of marrow cells dramatically decreased when the normal and the ablated femurs were subsequently transplanted into the back of recipient mice. Interestingly, we also demonstrated that BMP-2 induced bone formation in inverse proportion to the number of marrow cells in the bone marrow [25]. Therefore, in order to examine the relationship between FGF-2-induced bone formation and marrow cells in the marrow cavity, bone marrow ablation and transplantation experiments were performed. Contrarily to the result observed with BMP-2 [25], the bone formation induced by FGF-2 was dramatically inhibited in the normal femurs and the ablated femurs subsequently transplanted into the back of recipient mice (Figure 3). Taken together, these results and our previous report strongly indicate that bone marrow cells play important roles in the regulation of the activity of BMP-2 and FGF-2 in inducing bone formation.

### 2.4. Effects of BMP-2 and FGF-2 on Osteoblast and Osteoclast in Mouse Calvarial and Femoral Defects

**Figure 4:**
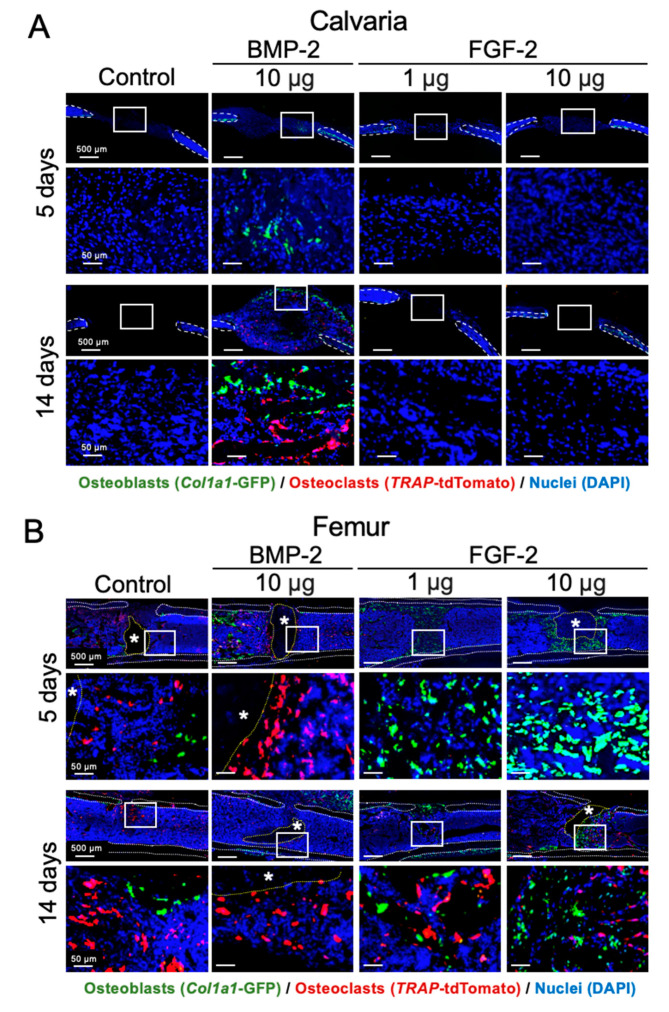
Effects of BMP-2 and FGF-2 on osteoblast and osteoclast in mouse calvarial and femoral defect. Cross-sectional frozen sections of calvaria (frontal plane, **A**) and femurs (**B**) obtained from *Col1a1*(2.3)-GFP/*Trap*-tdTomato mice after 5 and 14 days of collagen pellet implantation. GFP-positive osteoblasts and tdTomato-positive osteoclasts are shown in green and red, respectively. Dashed lines indicate the cortical bone (white) and the remained collagen pellet (*, yellow). Nuclei were stained with DAPI (blue). The results are representative of at least three independent experiments.

*Col1a1*(2.3)-GFP/*Trap*-tdTomato mice were used to understand the effects of BMP-2 and FGF-2 on osteoblasts and osteoclasts in vivo, using the calvarial and femoral defects. In the bone marrow-scarce calvaria, after 5 days of transplantation, BMP-2 promoted a remarkable migration of GFP-positive osteoblasts to the transplanted site. After 14 days, tdTomato-positive osteoclasts were also observed in the regenerated bone together with GFP-positive osteoblasts, indicating a remodeling of the over-formed bone induced by BMP-2. On the other hand, since the calvarial defect does not heal spontaneously, the two cell types were not observed in the control group and FGF-2 groups (Figure 4A).

In the bone marrow-abundant femur, however, FGF-2 promoted bone formation by attracting the GFP-positive osteoblasts to the healing site at 5 days post-transplantation. After 14 days, both GFP-positive osteoblasts and tdTomato-positive osteoclasts, which would be involved in the bone remodeling process, were observed at the transplanted site (Figure 4B). GFP-positive osteoblasts and tdTomato-positive osteoclasts were also observed in the control group, because the repair of bone defects in the femur occurs physiologically within 14 days. On the other hand, interestingly, in the group transplanted with BMP-2, only tdTomato-positive osteoclasts were observed at the transplanted site either at 5 days or 14 days post-transplantation.

### 2.5. Effects of BMP-2 and FGF-2 on Angiogenesis in Mouse Calvarial and Femoral Defects

**Figure 5:**
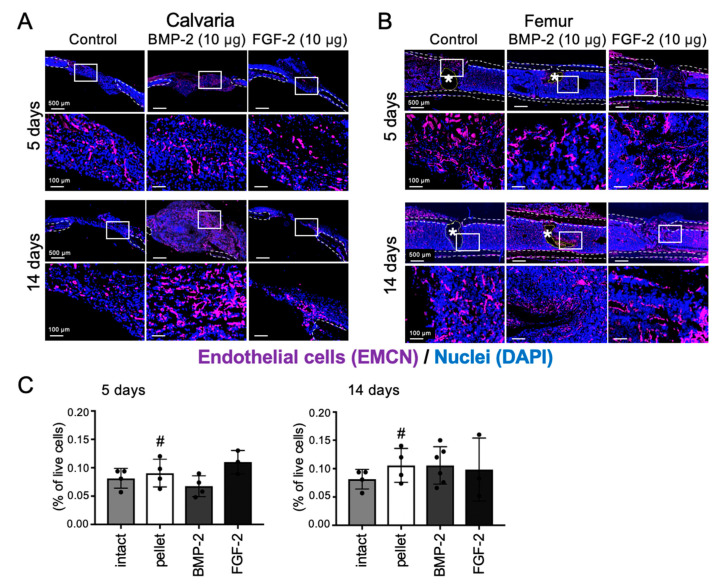
Effects of BMP-2 and FGF-2 on angiogenesis in mouse calvarial and femoral defect. Cross-sectional frozen sections of calvaria (frontal plane, **A**) and femurs (**B**) obtained from wild-type mice after 5 and 14 days of collagen pellet implantation. EMCN-positive endothelial cells are shown in purple. Dashed lines indicate the cortical bone (white) and the remained collagen pellet (*, yellow). Nuclei were stained with DAPI (blue). The results are representative of at least three independent experiments. Collagen pellets containing BMP-2 (10 µg) or FGF-2 (10 µg) or DW (control) were transplanted into mouse femur defects and samples were analyzed by FCM (**C**) at 5 days and 14 days after transplantation. Endothelial cell: CD31⁺CD45⁻. (*n* = 3–6, One-way ANOVA/Tukey).

Angiogenesis is a key process in the early period of bone healing, and both FGF-2 and BMP-2 have been reported to promote angiogenesis [27,28,29,30]. Therefore, to evaluate the effects of BMP-2 and FGF-2 on neovascularization in mouse calvarial and femoral defects, we performed IHC analysis for the detection of endomucin (EMCN), which is specifically expressed in the vascular endothelial cells of adult mice, and flow cytometric analysis (FCM) for detection of CD31^+^/CD45^−^ endothelial cells. The results showed that in the bone marrow-scarce calvaria, EMCN-positive endothelial cells were observed at the transplanted site in all groups, but remarkably in the regenerated bone induced by BMP-2, at 14 days post-transplantation (Figure 5A).

Oppositely, in the bone marrow-abundant femur, the number of EMCN-positive endothelial cells in the transplanted site was decreased in the BMP-2-transplanted group, whereas a high amount of the cells was observed in the control and FGF-2-transplanted groups at 5 days post-transplantation (Figure 5B). Next, FCM analysis was performed to calculate the number of CD31^+^/CD45^−^ endothelial cells. As a result, after 5 days of transplantation, the number of CD31^+^/CD45^−^ endothelial cells in the BMP-2- group and FGF-2-transplanted group showed a decreasing and increasing tendency, respectively. However, after 14 days, there was no difference in the number of CD31^+^/CD45^−^ endothelial cells between the control and experimental groups (Figure 5C).

### 2.6. Effects of BMP-2 and FGF-2 on Bone Marrow Cell Populations

**Figure 6:**
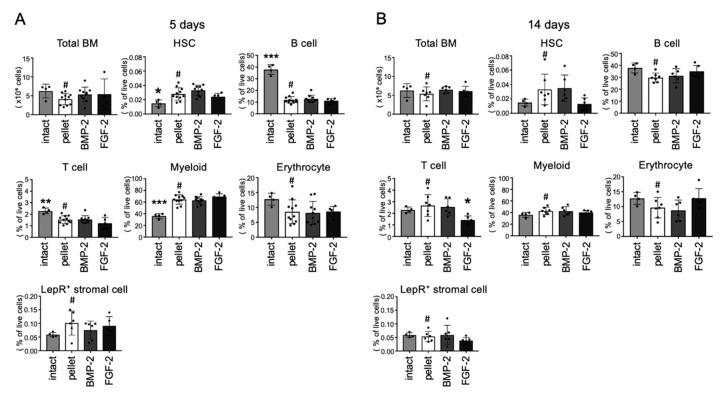
Effects of BMP-2 and FGF-2 on bone marrow cell populations. Collagen pellets containing BMP-2 (10 µg) or FGF-2 (10 µg) or DW (control) were transplanted into mouse femur defects and samples were analyzed by FCM at 5 days (**A**) and 14 days (**B**) after transplantation. HSCs: Lin^−^Sca-1^+^c-Kit^+^CD150^+^CD48^−^, Erythroid cells: Ter119^+^, Myeloid cells: CD11b^+^Gr-1^+^, B cells: B220^+^, T cells: CD3^+^, Leptin R^+^ stromal cells: CD45⁻Ter119⁻CD31⁻LepR⁺ (*n* = 4–11, * *p* < 0.05, ** *p* < 0.01, *** *p* < 0.001 versus control pellet (#). One-way ANOVA/Tukey).

Finally, the effect of both BMP-2 and FGF-2 on hematopoietic cell populations were analyzed by FCM. As shown in Figure 6A,B, bone marrow cellularity did not significantly change by application of the two growth factors. Note that the FGF-2-transplanted group displayed a significantly lower amount of T cell population (CD3^+^) than the group transplanted with the control pellet at 14 days post-transplantation. However, the percentage of live cells in the hematopoietic stem cell (HSC, Lin^−^Sca-1^+^c-kit^+^CD150^+^CD48^−^), B cell (B220^+^), erythrocyte (Ter119^+^) myeloid cell (CD11b^+^Gr-1^+^) populations were not significantly altered either by FGF-2 or BMP-2 (Figure 6A,B). In addition, BMP-2 and FGF-2 induced no significant change in the number of LepR positive stromal cells, which are known to maintain the hematopoietic stem/progenitor cell niche in the bone marrow (Figure 6A,B). Together, these results indicate that BMP-2 and FGF-2 have opposing effects on osteoblasts and osteoclasts in a bone marrow-abundant or scarce environment, but they induce no significant changes in the hematopoietic cell population or the stem cell niche.

## 3. Discussion

Bone regeneration therapy is one of the main topics of concern in regenerative medicine, and, in recent years, both BMP-2 and FGF-2 have been widely used for alveolar bone regeneration in the dental field. However, it is still unclear which growth factor should be recommended for alveolar bone regeneration. Most studies have shown that BMP-2 has a higher osteogenic ability than FGF-2 [16]. Additionally, complications associated with the application of the two growth factors have been increasingly reported. Therefore, the exact effects of the two growth factors on different cell types, including not only osteoblasts, osteoclasts but also bone marrow cells, should be investigated in more detail to promote more effective delivery of FGF-2 and BMP-2 to patients. In the present study, we evaluated the effects of BMP-2 and FGF-2 on bone formation in bone marrow scarce or abundant environments and found that BMP-2 had a high bone formation ability in the marrow-scarce calvaria, but induced bone resorption in bone marrow-abundant femurs. On the other hand, FGF-2 showed opposite effects compared to those of BMP-2. Moreover, bone marrow ablation and transplantation analysis revealed that BMP-2 and FGF-2-induced bone formation was regulated by the bone marrow cells in the marrow cavity (Figure 3) [25].

In accordance with these findings, we have recently demonstrated that BMP-2 has opposite functions in inducing, respectively, bone formation or resorption in a bone marrow-scarce or abundant environment. For instance, BMP-2 was shown to promote bone regeneration and osteointegration around titanium implants inserted in tooth extraction sockets (extramedullary environment), but induced bone resorption when the implant was inserted in the mandible marrow. Moreover, we showed that the BMP-2-induced bone resorption in the femur was regulated by a direct cell-cell interaction between the marrow cells and osteoblasts *in vitro* [25].

The results of this study are also in line with those reported by Kawaguchi et al., who showed that local administration of FGF-2 in long bone fractures, which are bone marrow-abundant sites, significantly accelerated fracture healing and callus formation [31]. Nagayasu et al. also demonstrated that local administration of FGF-2 in the bone marrow environment accelerated bone formation around the dental implant and osteointegration in a dog model [26].

At the cellular level, BMP-2 is known to stimulate the expression of the mineralization-associated genes but seems to have little or no effect on the expression of genes associated with cell proliferation [32]. FGF-2, on the other hand, seems to have different effects on bone-related cells depending on the cell differentiation stage. For instance, in mesenchymal progenitor cells, FGF-2 is known to be a strong inducer of the expression of genes associated with cell proliferation and expression of angiogenic genes, such as vascular endothelial growth factor A [32,33]. Moreover, the effects of FGF-2 on BMP-2 signaling seem to be dose-dependent; while a low dose of FGF-2 could enhance the BMP-2-associated bone formation, a high dose of FGF-2 suppressed it. Of note, FGF-2 has been reported to significantly reduce the expression of BMP-2 and alkaline phosphatase and the mineralization of osteoblasts [32]. FGF-2 also inhibited the phosphorylation of Smad-1 and increased the expression of the BMP-2 inhibitor Noggin, thus antagonizing the BMP cascade [34,35]. Together, despite the controversies in the literature, the opposite effects of FGF-2 and BMP-2 observed in the bone marrow-abundant and scarce sites could be directly or indirectly associated with their antagonistic interactions.

Bone is considered as a rigid organ that supports and protects various vital organs in the body, including the bone marrow and the stem cell niche [36]. In this context, BMPs are known to play a fundamental role in the development of hematopoietic bone marrow [37,38]. Devorah et al. reported that BMP-4, which is also important for both prenatal and post-natal bone formation and regeneration, is a critical component of the hematopoietic microenvironment that regulates both the number and function of HSCs [39]. Itkin et al. also reported that systemic administration of FGF-2 regulates in vivo expansion of both HSCs and their supportive stromal cells, such as CXCL12-abundant reticular (CAR) cells [40] and LepR positive stromal cells [41,42]. From these reports, we assumed that the transplantation of BMP-2 and FGF-2 in bone marrow could cause changes in the population of bone marrow cells. Contrary to our expectations, however, no significant change in the cell population of murine bone marrow, including LepR positive stromal cell, was observed by local administration of the two factors, indicating that the local delivery of the two factors is relatively safe. The difference in the systemic versus local delivery of the growth factors could be a major reason for the discrepancy, though future studies, for example with different concentrations of the growth factors, are necessary to clarify these conflicting findings.

In summary, our study demonstrated that BMP-2 and FGF-2 could only induce bone formation, respectively, in the bone marrow-scarce and abundant environments. Therefore, BMP-2 could be suitable for application in extramedullary, while the FGF-2, in medullary bone regeneration. Moreover, both BMP-2 and FGF-2 administrated in the bone marrow did not significantly affect the hematopoietic cell population, indicating a relatively safe application of the two growth factors. Finally, the two growth factors could be applied not only in the reconstruction or regeneration of large bone defects but also in combination with biomaterials, such as to allow a rapid osteointegration and long-term stability of implants, which are the key factors for successful functional rehabilitation in dental and orthopedic treatments.

## 4. Materials and Methods

### 4.1. Materials

Recombinant human BMP-2 derived from *Escherichia coli* was prepared using the methods reported previously [43,44]. FGF-2 (Fiblast Spray) was purchased from Kaken Pharmaceutical (Tokyo, Japan). In order to prepare FGF-2 and BMP-2 collagen pellets, 30 μL of atelocollagen (Koken, Tokyo, Japan) were mixed with BMP-2 (10 μg) or FGF-2 (1 and 10 μg). The control pellet was prepared by mixing atelocollagen with DW. After mixing, the pellets were frozen at −80 °C and dried in vacuum. The freeze-dried collagen pellets containing the growth factors were transplanted in the bone defects.

### 4.2. Animal Experiments

Eight-week-old c57BL/6 J mice were purchased from CLEA Japan (Tokyo, Japan). *Col1a1*(2.3)-GFP mice [45] and *Trap*-tdTomato mice [46] were kindly gifted from Dr. Matsuo (Keio University, Tokyo, Japan) and Dr. Ishii (Osaka University, Osaka, Japan), respectively. For all experiments, mice at 8–12 weeks of age were used. The animal experiment protocol used in this study (OKU-2019254) was approved by Okayama University Animal Research Committee. All animals were handled according to the guidelines of the Okayama University Animal Research Committee.

Collagen transplantation into the bone marrow-abundant femoral defect and the bone marrow-scarce calvarial defect was performed according to our previously reported methods [25]. Briefly, a bone defect was created at a distance of 3 mm above the knee in the mouse both femurs of mice by a 25-gauge needle and a collagen pellets containing DW (control), BMP-2 or FGF-2 were randomly transplanted into the marrow cavities of both femurs under general anesthesia. In calvaria, collagen pellets were transplanted into a 2 mm bone defect made by a biopsy punch (Kai Medical, Tokyo, Japan). To ablate bone marrow cells, femurs were drilled by a dental reamer and washed out with a phosphate buffer solution (PBS), and/or further translocated into the dorsal region of recipient mice.

### 4.3. Micro-CT Analysis

Fourteen days after transplantation, the collected femurs and calvaria were fixed with 4% paraformaldehyde (PFA) and analyzed by micro-computed tomography (micro-CT, SkyScan 1174, Aartselaar, Belgium) as described previously [25]. Bone volume/tissue volume (BV/TV) in the femur was analyzed at an area within 1.5 mm above and below the defect, and the regenerated bone volume (RBV) in the calvaria was analyzed using SkyScan software (NRecon, CTAn, CTvol, and CTvox, SkyScan).

### 4.4. Histological Analysis

Samples decalcified with Morse solution (Shiyaku, Kyoto, Japan) were sectioned and stained with hematoxylin and eosin (HE). For observation of osteoblast and osteoclast in *Col1a1*(2.3)-GFP/*TRAP*-tdTomato mice and immunohistochemical (IHC) analysis, frozen sections were prepared by using the Kawamoto’s film method, according to a previous report [47]. For IHC analysis, sections were blocked with 5% goat serum (Life Technologies, Gaithersburg, MD, USA) for 30 min at room temperature (RT) and incubated with anti-endomucin antibody (EMCN: sc-65495, Santa Cruz Biotechnology, Dallas, TX, USA) overnight at 4 °C. Sections were incubated with the secondary antibody, Alexa Fluor 647 goat anti-rat IgG (Life Technologies) for 1h at RT in a dark chamber. All images were taken by a BZ-710 fluorescence microscope (Keyence, Osaka, Japan).

### 4.5. Flow Cytometry

For flow cytometric (FCM) analysis, bone marrow cells were flushed out from mouse femur with PBS containing 2% fetal bovine serum (FBS, 2% FBS/PBS) and gently dissociated by passing the cells through a 21-gauge needle. Finally, the cells were suspended in 2% FBS/PBS and stained with fluorescent-labeled antibodies (Table 1). FCM analyses were carried out using the MACSQuant (Miltenyi Biotec, Bergisch Gladbach, Germany) and data analysis was performed with FlowJo (BD Biosciences, San Jose, CA, USA). Cell populations were identified as follows: HSC: Lin^−^Sca-1^+^c-Kit^+^CD150^+^CD48^−^, B cell: B220^+^, T cell: CD3^+^, Myeloid cell: CD11b^+^Gr-1^+^, Erythrocyte: Ter119^+^, Leptin receptor (LepR) positive stromal cell: CD45^−^Ter119^−^CD31^−^LepR^+^, Endothelial cell: CD45^−^CD31^+^.

## 5. Statistical Analysis

The results obtained from quantitative experiments were reported as the mean values ± SD. Statistical analyses were performed with one-way factorial ANOVA followed by Tukey’s multiple comparison tests.

## Figures and Tables

**Table 1:** Antibodies used for flow cytometry.

Antibody	Conjugation	Clone	Source	Cat.#
CD3e	APC	145-2C11	BioLegend	100312
CD11b	APC	M1/70	BioLegend	101212
CD16/32		2.4G2	BD Biosciences	553142
CD31	APC	MEC13.3	BioLegend	102509
CD31	PE	MEC13.3	BioLegend	102507
CD45	APC	30-F11	BioLegend	103111
CD45R (B220)	APC	RA3-6B2	BioLegend	103211
CD48	BV421	HM48-1	BioLegend	103428
CD117 (c-Kit)	FITC	2B8	BioLegend	105806
CD150	PE	TC15-12F12.2	BioLegend	115903
Leptin Receptor	Biotin		R&D systems	BAF497
Lineage marker cocktail	APC	145-2C11(CD3e), M1/70(CD11b), RA3-6B2 (CD45R/B220), TER-119 (Ly76), RB6-8C5 (Ly6G/C)	BD Biosciences	51-9003632
Ly-6A/E (Sca-1)	PE-Cy7	D7	BioLegend	108114
Ly-6G/C (Gr-1)	BV421	RB6-8C5	BD Biosciences	562709
Streptavidin	BV421		BioLegend	405226
Ter119	APC	TER-119	BioLegend	116211
7-AAD			BioLegend	420404
